# Protocol for the development and validation of a driving simulator for evaluating the influence of drugs on driving performance

**DOI:** 10.1097/MD.0000000000014613

**Published:** 2019-02-22

**Authors:** Mari Iwata, Kunihiro Iwamoto, Tomohiro Omura, Masahiko Ando, Norio Ozaki

**Affiliations:** a Department of Psychiatry, Nagoya University, Graduate School of Medicine, Nagoya, Aichi; b Taisho Pharmaceutical Co., Ltd., Tokyo; c Center for Advanced Medicine and Clinical Research, Nagoya University Hospital, Nagoya, Aichi, Japan.

**Keywords:** Alcohol, ALDH2, driving performance, driving simulator, validation

## Abstract

**Introduction:**

Although automobile driving is often necessary in daily life, most package inserts for psychotropic drugs in Japan prohibit patients from driving under the influence of medication. This may be partially because no system to evaluate the influence of drugs on driving performance has been established. Standardized evaluation methods have been established in the Netherlands and the United States, but these cannot be implemented in Japan because of differences in road situations, traffic laws, and ethnicities. Therefore, to establish a method to evaluate the influence of drugs on driving performance in Japan, we planned a validation study using alcohol and a driving simulator (DS) and set a clinically meaningful threshold involving the standard deviation of lateral position (SDLP), which is a criterion standard evaluation item.

**Methods:**

This study was designed as a double-blind, placebo-controlled, randomized, 4-way, fourth-order crossover trial (Williams design). Twenty-four healthy Japanese men aged 21 to 64 years will be recruited through advertisements. The participants will be required to drive daily for over 3 years and to carry the active-type aldehyde dehydrogenase (ALDH) gene polymorphism (ALDH 2∗1/∗1). Participants will be randomly assigned to 4 groups based on blood alcohol concentration (BAC): 0% (placebo), 0.025%, 0.05%, and 0.09%. The amount of alcohol intake will be calculated based on Widmark formula using a beverage that is a mixture of 40% vodka and orange juice. After a practice period, each examination period will be set with 6-day intervals. The primary outcome is SDLP in a 60-minute road-tracking test using the DS. The secondary outcomes are other evaluation items in the DS tasks and DS sickness and sleepiness according to questionnaire responses. The estimated difference in SDLP between BAC levels of 0.05% and 0% will be calculated using a linear model.

**Ethics and dissemination:**

Ethics approval was obtained from the Ethics Committee at Hakata Clinic and the Nagoya University Medical School Hospital Bioethics Review Committee. The trial results will be disseminated through peer-reviewed publications and international conferences.

**Trial registration:**

This study was registered at ClinicalTrials.gov NCT 03572985 on June 28, 2018.

## 1 Introduction

Automobile driving is an important means of transportation in modern society and an indispensable everyday activity for many people who live outside of large cities where no well-organized public transportation is available. This is also true for patients with mental disorders that require continuous medication to improve symptoms and prevent relapse. However, the World Health Organization has published a policy brief for drug use and road safety indicating that the effects of prescription drugs could not be underestimated.^[1]^ In addition, the US Food and Drug Administration has asked pharmaceutical companies to examine the influence of drugs that affect the central nervous system on driving.^[2]^ In Japan, although conclusive evidence is lacking, almost all the package inserts for psychotropic drugs prohibit patients from driving under the influence of medication. These uniform regulations restrict patients’ daily lives.

One of the reasons for this situation is that no system has been established in Japan for evaluating the influence of drugs on driving performance. Despite considerable research on driving performance in many countries, each research facility uses different evaluation methods, such as actual vehicle tests and driving simulators (DSs).^[3]^ Recently, results regarding the effect of sleeping pills on driving performance have been considered in devising recommended clinical dosages^[4]^; however, only the evaluation system in the Netherlands using actual cars^[5]^ and that in the United States using DSs^[6]^ is used in drug approval applications. In particular, the evaluation of vehicle “weaving” in the lateral direction became an index of driving performance referred to as the standard deviation of lateral position (SDLP), and currently, this remains the only fully validated index.^[3]^ Although these evaluation systems are accepted as standard methods abroad, the traffic laws and road conditions in foreign countries differ considerably from those in Japan. Moreover, the results obtained by those evaluation systems can be affected by ethnic differences, especially the evaluation system in the Netherlands, which has been verified only on long-distance linear expressways; therefore, using the same evaluation methods across countries and cultures is difficult. The establishment of a system in Japan that evaluates the influence of drugs on driving performance could provide useful information for Japanese patients and physicians.

Therefore, with the aim of establishing such an evaluation system, we planned a validation study using a DS and alcohol that would meet legal standards around the world. The purpose of this study targeting healthy adult males is to set a clinically meaningful SDLP threshold as a main evaluation item based on the difference between SDLP at blood alcohol concentration (BAC) levels of 0.00% and 0.05%. We set the SDLP threshold with a BAC of 0.05% as the impaired level. The upper limit of BAC set by law ranges from 0% to 0.08% from country to country.^[7]^ However, the International Council on Alcohol, Drugs, and Traffic Safety^[8]^ reported a BAC <0.05% is relatively safe, a BAC of 0.05% to 0.08% induces mild to moderate side effects, and a BAC of ≥0.08% poses a potentially dangerous risk. In fact, in most countries, a BAC of 0.05% is set as the upper limit. Although some reports have shown that cooperative muscle movement is impaired starting at a BAC of 0.035%,^[9]^ epidemiological studies have indicated that the risk of being involved in a traffic accident increases only slightly at BAC levels of ≤0.04%,^[10]^ increases by about double around a BAC level of 0.05%, and increases exponentially starting at a BAC level of 0.10%.^[11]^ Therefore, for the purposes of this study, we decided to use a BAC level of 0.05% as a threshold for clinically meaningful driving impairment. As a BAC level of ≥0.10% can easily cause drunkenness, 4 BAC levels (0.00% [placebo], 0.025%, 0.05%, and 0.09%) will be set to calculate the SDLP regression curve. The hypothesis of this study is that SDLP will increase in accordance with BAC levels.

## 2 Methods

### 2.1 Study design

This study was designed as a double-blind, placebo-controlled, randomized, 4-way, fourth-order crossover trial (Williams design). It will be an intervention study (alcohol intake and DS operation) with no drug administration. Taisho Pharmaceutical Co., Ltd., will be conducting the clinical trial at Fukuoka Mirai Hospital in Japan. To allow the participants to become accustomed to operating the DS, a practice period involving the same contents as the examination period will be established. The practice period will be conducted during a 2-day/1-night hospitalization stay and the examination period during a 3-day/2-night stay. A 6-day interval period will be implemented between the practice and examination periods.

### 2.2 Participants

Healthy Japanese men were recruited through online advertisements and from Fukuoka Mirai Hospital. Because this is an exploratory study, the sample size was set to 24 participants in reference to the sample size of previous studies conducted to confirm the validity of a DS.^[6,12]^ The inclusion criteria are: age between 21 and 65 years; body mass index between 18.5 and 24.9 kg/m^2^; active-type aldehyde dehydrogenase (ALDH) gene polymorphism (ALDH 2∗1/∗1); alcohol consumption >2 days a month; able to drink a prespecified amount of alcohol in 30 minutes; possession of a driver's license and driving daily for >3 years; consistent sleeping pattern (wake up between 06:00 and 09:00 am, go to bed between 21:00 and 00:00 pm); no visual impairments; able to operate a DS with a full understanding of all DS tasks; judged by a physician as being able to participate; and able to provide written informed consent before the examination begins. The exclusion criteria are: having a disease recognized as being nonhealthy by a physician; a history of drug or food allergies; 3) serious allergic predispositions; 4) a history of stroke, head trauma, epilepsy, or malignant tumor; more than a 3-month history of sleep disorders, a medical history of sleep apnea syndrome or restless legs syndrome, or a history of hypersomnia such as narcolepsy; use of over-the-counter drugs within 1 week after starting the practice period; use of sedative hypnotics within 4 weeks after starting the practice period; experiencing more than a 6-hour time difference within 4 weeks after starting the practice period; irregular shift work and night shift work within 4 weeks after starting the practice period; experience using the same DS evaluation method as that used in the present study; a daily routine of alcohol consumption until sleep; unable to stop drinking from 2 days before until the day of the screening test, and from 2 days before hospitalization until discharge; smoking during hospitalization; donating blood within 12 weeks; use of prescription drugs within 4 weeks after starting the practice period; a diagnosis or history of alcoholism or drug dependency; positive result from a urine drug test during screening; unable or unwilling to comply with the study protocol; and judged unsuitable for participation by a physician. The discontinuance criteria are: noncompliance with the study protocol; experiencing adverse events that compel a physician or the participant himself/herself to cease participation in the trial; the participant chooses to discontinue the trial of their own volition; sliding off the track or have a large SDLP such as >60 cm during the practice period; and judged unsuitable for participation by a physician.

### 2.3 Randomization and blinding

In total, 24 registered participants will be assigned to the following 4 sequences based on BAC: 0%, 0.025%, 0.05%, and 0.09%. The participants will be assigned randomly so that the ratio of each sequence will be 1:1:1:1 (Table 1). Randomization will be conducted based on a computer-generated random number table, with allocation conducted by an assignment manager uninvolved in data collection and not disclosed until the BAC groups are fixed. Therefore, the participants and investigators will be blinded to the allocation. However, in addition to the assignment manager, the allocations are planned to be disclosed to the institution for BAC measurement.

**Table 1:**
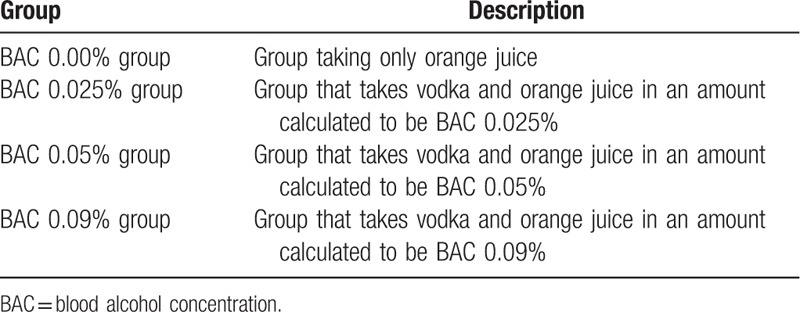
Allocation of the participants into groups based on BAC.

### 2.4 Determination of alcohol intake

In previous studies, the amount of alcohol intake has been calculated individually based on body water content.^[12,13]^ Therefore, we decided to calculate the amount of alcohol intake based on Widmark formula using a beverage that is a mixture of 40% vodka and orange juice. Using this formula, tests are conducted in the morning and afternoon, so the following equation will be used: 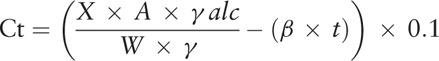


The variables used in Widmark formula are defined in Table 2.

**Table 2:**
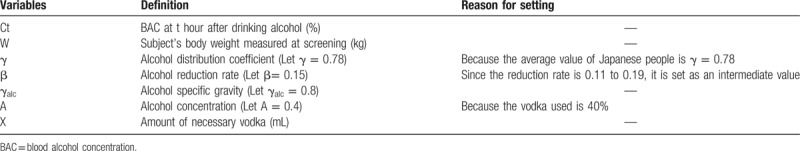
Definition of variables used in Widmark formula.

The average of the BAC measured before and after the DS evaluation will be taken as the BAC at the DS evaluation.

- BAC calculation formula in the morning: X = (Cm × 10 + 0.15) × W × 2.44 (mL)
- BAC calculation formula in the afternoon: X = (Cm × 10 + 0.089) × W × 2.44 (mL)

### 2.5 ALDH2 genotype test

In this study, the ALDH2 genotype test will be performed by blood sampling at the screening stage. Although previous studies conducted outside of Japan have carried out DS validation using alcohol,^[14]^ generally, Asians, including Japanese, cannot drink as much alcohol as Europeans and North Americans; therefore, careful attention is needed in experimental designs targeting Japanese populations. Almost 100% of Caucasoids have the *ALDH2 ∗ 1* gene, which is associated with enhanced alcohol metabolism,^[15]^ whereas about 10% to 60% of Mongoloids have the *ALDH 2 ∗ 2* gene, which is associated with low enzyme activity.^[16]^ In the case of *ALDH 2 ∗ 2*, the accumulation of aldehydes may affect the results, making them difficult to compare with those of previous studies. Therefore, to check whether the participants had the *ALDH 2 ∗ 1* gene, all patients will undergo a blood sampling test before the DS task.

### 2.6 Experimental schedule

**Figure 1:**
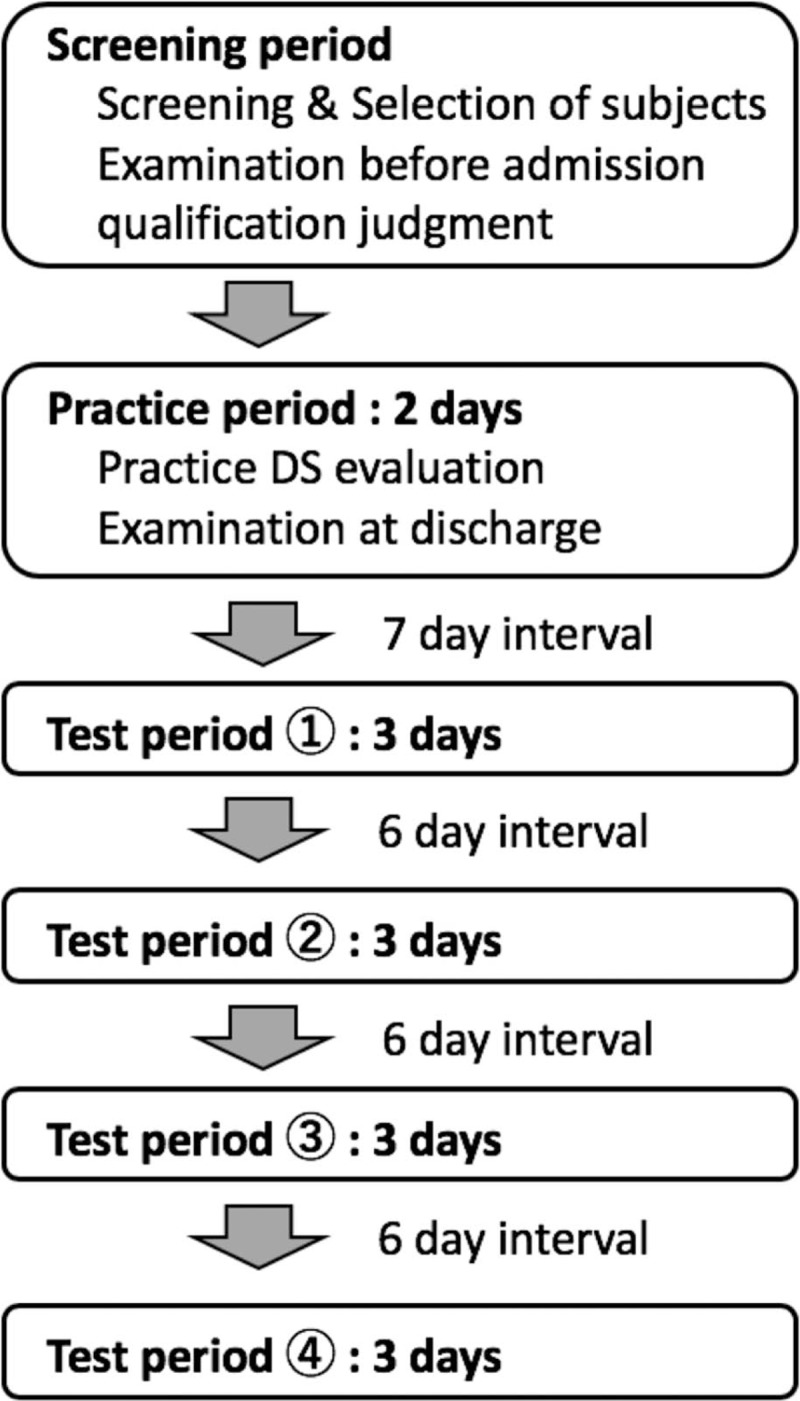
Flowchart of the experiment. DS = driving simulator.

A flowchart of the experiment is shown in Figure 1. The examination is divided into a screening period, a practice period, and 4 test periods. The interval between the practice period and test period 1 is 7 days, and the interval between each test period is 6 days. All participants will undergo screening tests, including assessment of background characteristics, a medical examination, check of vital signs, electrocardiogram, hematological examination, urine drug test, ophthalmic examination, and genetic test for ALDH2 during the screening period. The medical examinations and vital sign checks will be conducted at the time of hospital admission (Days 1, 8, 16, 24, and 32) and discharge (Days 1, 10, 18, 26, and 34) and before the DS task (Days 1, 9, 17, 25, and 33) during the practice and test periods. The time schedule of the test period is shown in Table 3. Participants will be given a chance to become accustomed with operating the DS on the evening of the first day of admission (days 1, 9, 17, 25, and 33) during the practice and test periods. They will be admitted to the hospital the day before the DS task and subjected to DS experiments the day after admission; all participants will be discharged from the hospital at 3 days after admission after their safety has been confirmed. All participants will also be evaluated using the Simulator Sickness Questionnaire (SSQ) before and after the DS task on Day 1, the Karolinska Sleepiness Scale (KSS), and the Profile of Mood States (POMS) 2 before the DS task on days 1, 9, 17, 25, and 33. Blood sampling for BAC measurements will be conducted before and after the DS task on Days 9, 17, 25, and 33.

**Table 3:**
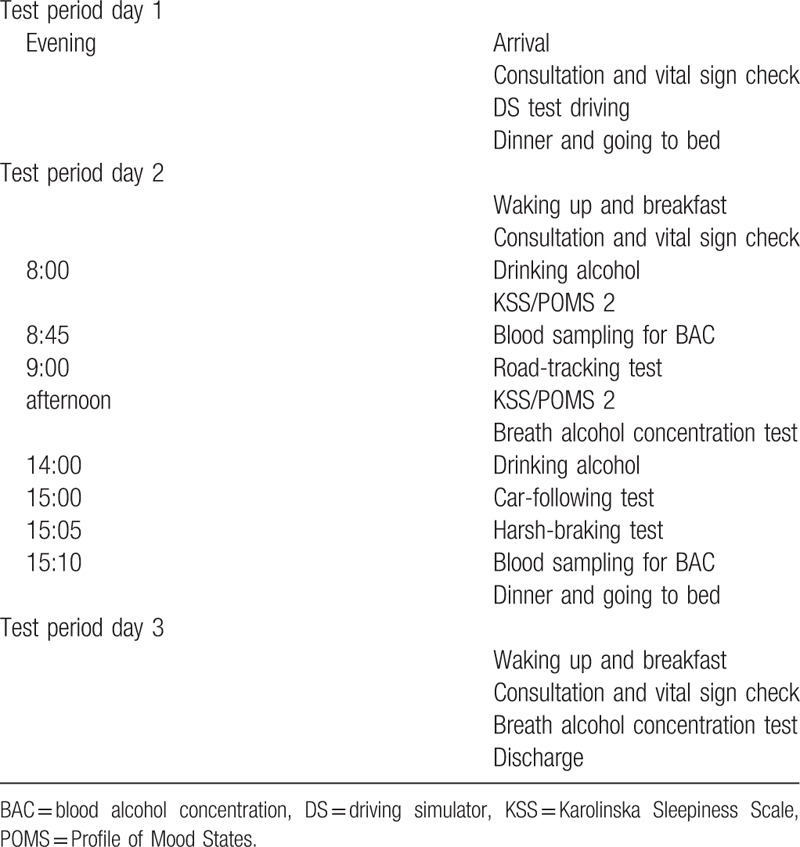
Time schedule of the test period.

### 2.7 DS task

The DS software runs on a PC (Windows 10) equipped with a steering wheel, brake pedal, and accelerator system (Driving Force GT, Logicool). The image from the PC is projected on an 80-inch screen using a liquid crystal projector (EB-X05, Epson, Nagano, Japan). The DS tasks consist of the following. In a road-tracking test, the participants are instructed to drive in the center of the left lane while maintaining a speed of 100 km/h on a 2-lane road with gentle curves. The SDLP, which indicates weaving, is used as a performance measure. The measurement time is 60 minutes from 9:00 or 10:15 on the DS evaluation day. In a car following test, the participants are instructed to maintain a constant intervehicle distance from a preceding vehicle with varying speeds. When the preceding car decelerates, its brake lights come on. Performance is measured as the coefficient of variation (CV), which is obtained by dividing the standard deviation of the distance between the cars by the mean value. Therefore, a smaller CV of the distance to the preceding vehicle (DCV) indicates better performance. The measurement time is 5 minutes from 15:00 or 16:15 on the DS evaluation day. In a harsh braking test, the participants are instructed to maintain a constant speed of 50 km/h and to avoid colliding with humanoid models randomly appearing from either side of the road by harsh braking. This test consists of seven harsh braking trials over a 5-min period, and is conducted continuously after the completion of the car following task. The DS tasks are conducted in a dark, sound-attenuated room and based on our previous DS tasks.^[17]^ Driving performance is recorded every 20 ms.

### 2.8 Primary outcome

The primary outcome is SDLP (in centimeters), which is the distance from the center line of the road to the right end of the car body in the road tracking test. SDLP in the road tracking task known to be a more sensitive index than other variables,^[18]^ and its validity and reliability have been confirmed.^[5,11,19]^ Since SDLP has been used as a primary evaluation item to assess the influence of drugs on driving performance, it was also set as the main evaluation item in the present DS study.

### 2.9 Secondary outcomes

The following secondary DS outcomes will be used: total number of times the car body crosses the lane (inappropriate line crossing; ILC); total number of times the vehicle goes off of the course (course-outs); standard deviation of speed in the road tracking test; reaction time for detecting deceleration of the preceding vehicle (time to speed adaptation); number of collisions with the preceding vehicle (car collisions) in the car following test; brake reaction time (BRT); and number of collisions with an object (error) in the harsh braking test.

### 2.10 Other outcomes

In addition to the DS evaluation, the following items will also be evaluated. Considering the possibilities of DS sickness and drowsiness at the time of the examination and alcohol-induced mood changes affecting the results, the Japanese version of the KSS,^[20]^ Japanese version of the POMS 2,^[21]^ and SSQ^[22]^ will be conducted simultaneously.

### 2.11 Statistical analysis

For the statistical analysis, we will calculate the basic statistics of accumulated SDLP through 60 minutes for each BAC and the difference from BAC 0%. The predicted difference in SDLP between the BAC 0.05% and 0% groups will be calculated using a linear model. Basic statistics for the secondary outcomes will also be calculated. Evaluation items with incomplete data will be excluded from analysis, and outliers will be treated as missing values.

### 2.12 Adverse events

If any adverse events occur after the start of the practice period, depending on the severity, the test will be stopped based on a decision by the doctor or the participant himself/herself, and the problem will be treated appropriately. All adverse events will be reported at the end of the study and listed, but not aggregated or analyzed.

### 2.13 Ethics and dissemination

This study was registered at ClinicalTrials.gov (NCT 03572985) on June 28, 2018. The study protocol was approved by the Ethics Committee at Hakata Clinic (1747CP-3) and the Nagoya University Medical School Hospital Bioethics Review Committee (2010-0970-3), and the study has been being performed at Fukuoka Mirai Hospital. Informed consent was obtained from all study participants. For privacy protection, participants will be identified using an identification code. Information such as the participant's name and address will be managed only at the medical examination center and will not be provided to other organizations. If any necessary experimental data are provided to a joint research institution (sponsor and investigator), these will be carefully protected using only the participants’ identification codes and a corresponding table. The sponsor and investigator will have access to the final test data, and the final results will aim to be published in a journal article. The acquisition of informed consent, inclusion/exclusion criteria, participants’ eligibility, and occurrence of any adverse events will be confirmed by monitors from outside the testing agency. These monitors will ascertain whether the experiment is being carried out according to the approved procedure and confirm that the data storage method is appropriate. We will also set up an independent auditor from the testing department who evaluate whether the experiment complies with the protocol. All test-related data will be disclosed to the monitor or auditor for the purposes of conducting a survey. The findings will be aimed to be published in peer-reviewed journals and presented at local, national, and international conferences to publicize and explain the research to key audiences.

## 3 Discussion

This study will examine the validity of using a DS with alcohol to evaluate the influence of drugs on driving performance. To our knowledge, few evaluation systems have been validated using alcohol, so the present protocol represents the first time a DS will be used as the evaluation system in combination with alcohol in the Japanese population. DSs are less expensive and safer than actual vehicles, and thus allow more extensive research to be conducted and more appropriate information to be provided to doctors, pharmacists, users, and citizens.

As for existing DSs, including Cognitive Research Corporation's Driving Simulator (CRCDS Mini-Sim), the Systems Technology Inc. Simulator (STISIM), and that of the Würzburg Institute for Traffic Sciences (WIVW), validity verification using alcohol has been carried, but with differing methodologies. In the STISIM, the amount of alcohol consumed was determined individually according to separate protocol,^[23]^ and then the validity was verified using a 20-minute scenario involving highway driving with 4 BAC crossover sets (0.00%, 0.05%, 0.08%, and 0.10%).^[24]^ The CRCDS Mini-Sim measured SDLP in a road-tracking test after participants ingested an individually set amount of alcohol to reach a BAC of 0.10%,^[13]^ and that index was shown to be more sensitive than that of the STISIM.^[25]^ In WIVW research, the validity was verified by measuring SDLP and the number of errors in a road tracking test involving both highway and urban traffic scenarios using BAC crossover sets of 0.00%, 0.05%, and 0.08%.^[12]^

Although previous research has utilized many types of evaluation items individually, only SDLP has been validated as a main evaluation item.^[5,19]^ A correlation between SDLP and BAC values in a road tracking test has been reported in several studies, and a BAC of 0.05%, which is the legal driving limit in many countries, is known to correspond to an average increase in SDLP of about 2.4 cm in actual vehicle tests.^[11]^ However, SDLP values vary depending on the evaluation system, and it is generally known that DSs tend to increase SDLP compared with actual vehicle tests. To ensure the importance of this research, showing that the SDLP value increases with BAC will be indispensable. Another study reported that ILC, which represents the number of times SDLP increases above a certain level, is affected by alcohol consumption,^[25]^ so this will also be included as a secondary evaluation item in the present study.

DCV is an indicator that reflects attention and sensory functions during driving.^[26]^ As DCV is difficult to measure in actual vehicle tests, using a DS offers an advantage in terms of safety. Furthermore, rear-end collisions are the most common type of car accidents reported overseas and in Japan,^[27,28]^ which suggests that DCV measurements are likely to help predict accident risk. BRT is also an indicator that reflects attention and cognitive/behavioral functions during driving^[29]^; however, its validity has not been adequately confirmed, so it is set only as an exploratory evaluation item in this study. Although these exploratory items are complementary and the influence of alcohol has not been sufficiently verified, the results are expected to provide useful information in regard to the multilateral evaluation of driving skills.

A validated evaluation method for driving performance that is applicable in Japan would be extremely useful, as it could provide scientific verification of the influence of drugs on driving performance for use in prescription information and package inserts, thereby improving the information available to patients.

## Acknowledgments

The authors thank Iwao Kitajima and Ryuji Kuroishi of Taisho Pharmaceutical Co., Ltd. for help in developing the study protocol.

## Author contributions

NO developed the study concept with MI, KI, and TO. MI and KI wrote the first draft of the manuscript, and TO, MA, and NO provided critical revisions of the manuscript. All authors read and approved the final manuscript to be submitted.

**Conceptualization:** Mari Iwata, Kunihiro Iwamoto, Tomohiro Omura, Norio Ozaki.

**Funding acquisition:** Kunihiro Iwamoto, Norio Ozaki.

**Methodology:** Mari Iwata, Kunihiro Iwamoto, Tomohiro Omura.

**Supervision:** Masahiko Ando, Norio Ozaki.

**Writing – original draft:** Mari Iwata, Kunihiro Iwamoto, Tomohiro Omura.

**Writing – review & editing:** Masahiko Ando, Norio Ozaki.

Kunihiro Iwamoto orcid: 0000-0003-3868-3372.
